# Posturographic measures did not improve the predictive power to identify recurrent falls in community-dwelling elderly fallers

**DOI:** 10.6061/clinics/2020/e1409

**Published:** 2020-03-26

**Authors:** Kelem de Negreiros Cabral, Guilherme Carlos Brech, Angelica Castilho Alonso, Aline Thomaz Soares, Davi Camara Opaleye, Julia Maria D'Andrea Greve, Wilson Jacob-Filho

**Affiliations:** I Laboratorio de Estudos do Movimento, Instituto de Ortopedia e Traumatologia, Faculdade de Medicina (FMUSP), Universidade de Sao Paulo, Sao Paulo, SP, BR; II Divisao de Geriatria, Faculdade de Medicina (FMUSP), Universidade de Sao Paulo, Sao Paulo, SP, BR; III Programa de Ciencias do Envelhecimento, Universidade Sao Judas Tadeu (USJT), Sao Paulo, SP, BR; IV London School of Hygiene and Tropical Medicine, London, England

**Keywords:** Accidental Falls, Aging, Postural Balance, Risk Factors

## Abstract

**OBJECTIVE:**

This study aimed to evaluate if posturography can be considered a recurrent fall predictor in elderly individuals.

**METHODS:**

This was a cross-sectional study. A total of 124 subjects aged 60 to 88 years were evaluated and divided into two groups—the recurrent fallers (89) and single fallers (35) groups. Patients' sociodemographic characteristics were assessed, and clinical testing was performed. The functional test assessment instruments used were timed up and go test (TUGT), Berg Balance Scale (BBS), five times sit-to-stand test, and Falls Efficacy Scale (to measure fear of falling). Static posturography was performed in a force platform in the following three different situations—eyes open (EO), eyes closed (EC), and EO dual task.

**RESULTS:**

There were significant differences between the single and recurrent fallers groups regarding the fear of falling, the Geriatric Depression Scale score, the mean speed calculated from the total displacement of the center point of pressure (COP) in all directions with EO, and the root mean square of the displacement from the COP in the mediolateral axis with EC. Based on the hierarchical logistic regression model, none of the studied posturographic variables was capable of significantly increasing the power of differentiation between the recurrent and single fallers groups. Only TUGT with a cognitive distractor (*p*<0.05) and the BBS (*p*<0.01) presented with significant independent predictive power.

**CONCLUSION:**

TUGT with a cognitive distractor and the BBS were considered recurrent fall predictors in elderly fallers.

## INTRODUCTION

The incidence of falls increases with age. Approximately 30% of community-dwelling elderly adults worldwide experience a fall at least once a year 1. Among those, an estimated 15% will have recurrent falls, defined as two or more falls over a 12-month period 2. Recurrent events are significantly associated with high morbidity, an association that is more poorly understood compared to the occurrence of a first and isolated event 3,4.

The majority of falls result from a complex interaction of factors that can compromise the systems responsible for maintaining postural balance 5. These are associated with physiological changes of aging, pathological conditions, adverse effects of drugs, concomitant use of medications, environmental hazards, and inadequate behaviors 6. Falls have several adverse clinical outcomes including fractures, hospitalization, fear of falling, restriction of activities, and an increased risk of institutionalization 7-9.

In the Brazilian population, data from hospital admissions show that approximately 70% of injuries resulting from falls are bone fractures, specifically femoral fractures. The healthcare cost of fractures in elderly individuals increases annually 9. Data published by the Ministry of Health, which is available online at the Department of Health System Information, related to morbidity due to falls involving elderly residents in Brazil show that the hospital cost due to falls in elderly individuals was R$ 464,874,275.91 from 2005 to 2010 10. The relevance of both the adverse outcomes and costs of falls and the recurrence of these events should be paid careful attention. The risk factors for falls may have complex interactions that promote a vicious cycle of deteriorating health among the elderly.

A deteriorating postural balance is inherent in aging, and postural balance, defined as the capacity to maintain the center of gravity within the base of support, is considered fundamental for the efficient and safe execution of the activities of daily living (ADLs) 11. The preservation of postural balance contributes to the functional independence of the elderly and prevents falls, subsequently maintaining an individual's functional capacity 12. The use of posturographic parameters to determine falls among elderly individuals has been widely studied 13-17 considering, in part, that these parameters have sufficient information regarding an individual's postural control. Posturography is a technique used to measure bodily oscillations from a center point of pressure (COP). An increase in the average amplitude of displacement, the speed of displacement (VAvg), and an increase in the mediolateral displacement (XSD) of COP are associated with increased fall risk among elderly individuals 13,17,18. The force platform is an equipment used to measure COP, and the portable model (AMTI) is considered a reliable instrument for this purpose 19. Under these parameters, the evaluation using posturography has demonstrated a more objective and quantitative measure of equilibrium deficits 20 and has an important clinical use in evaluating fall risks among elderly individuals 15,. However, the role of posturography as an instrument used to help identify the recurrent fallers is still unclear, and determining whether posturography has a critical role in identifying the recurrent fallers is considered beneficial in properly allocating the available healthcare resources to prevent future falls.

A comprehensive understanding of the factors associated with recurrent falls and the role of posturography in discriminating the recurrence of falls among elderly fallers is significantly required in this study. Considering this knowledge gap, this study aims to evaluate if specific measures using posturography could be useful in differentiating recurrent from single fallers as part of a clinical evaluation.

## METHODS

### Study Location and Ethical Issues

The current study was part of a longitudinal study titled “Falls Prevention Program in the Older Individuals: Development, Implementation and Evaluation” (ClinicalTrials.gov Identifier: NCT01698580 24 (protocol number: 0145/11). It was conducted at the Motion Study Laboratory of the Institute of Orthopedics and Traumatology, Falls Prevention Program, Department of Geriatrics, Hospital das Clinicas, Medical School, Faculty of Medicine of the University of São Paulo and was approved by the hospital's ethics committee (approval number: 0145/11).

### Type of Study and Subjects

This was a cross-sectional study that used a convenience community-dwelling elderly sample based on the Falls Prevention Program of the PREVQUEDAS BRAZIL trial 24. It consisted of elderly patients who had fallen at least once for the past 12 months prior to the study. They were divided into two groups, the recurrent fallers group and the single fallers group. A recurrent faller was anyone who experienced a fall more than two times prior to the study.

### Measurements

The clinical evaluation consisted of a structured questionnaire used by the multidisciplinary health team trained on assessing the risk factors for falls, and static posturography was also used in this study 25,26. The average length of each assessment was 2 hours, and the sociodemographic characteristics collected from the study sample included age, sex, race, educational level, marital status, and family income.

Subjects were questioned about a fall event by asking “Have you experience falling for the past 12 months?,” considering a fall as an unintentional event that resulted in the change in the individuals' body position to a lower level, compared to their initial position, whether this fall was associated with adverse outcomes or not 27.

The subjects' health conditions were evaluated based on self-reported comorbidities (e.g., hypertension, diabetes, osteoporosis, osteoarthritis, major depression), the total number of medications (analgesics, antihypertensives, psychotropics) received, and subjects' behaviors (e.g., use of any psychotropic drugs, cognitive status [Mini-Mental State Examination, MMSE], depressive symptoms [Geriatric Depression Scale-15, GDS-15], visual acuity [Snellen test]).

The assessment of the subjects' functional mobility was performed using the timed up and go test (TUGT) with a cognitive distractor 28. It was performed under a dual-task paradigm, 29,30, a verbal fluency, through the backward evocation of the days of the week, time in seconds. Postural balance and muscle strength were assessed using the Berg Balance Scale (BBS) 31, consisting of 14 functional tasks with a total of 56 points, and the five times sit-to-stand test 32, time in seconds, respectively. Fear of falling was directly assessed by asking the question “Do you have a fear of falling again?” and indirectly assessed using the Falls Efficacy Scale-International (FES-I) 33,34, with a total maximum points of 64 (minimum points, 16).

The postural balance assessment (posturography) was performed on a portable force platform (AccuSway Plus^®^, AMTI1, MA, USA) 25,26. The data were collected and stored using the Balance Clinic software, configured to a frequency of 100 Hz with a cutoff frequency of 10 Hz. All subjects were tested while assuming a standardized position in relation to the maximum width of the support base (less than hip width), with arms beside the body and head looking straight at a target. Moreover, three measurements were obtained with the eyes open (EO), three with the eyes closed (EC), and three with dual tasking for 60 seconds each. The mean of the three assessments was calculated. The parameters used to measure the subject's stability were the root mean square of the displacement from the COP in the mediolateral axis (XSD) and the mean speed calculated from the total displacement (VAvg) of the COP in all directions.

### Statistical Analysis

The descriptive analysis was performed using the Statistical Package for the Social Sciences version 18 and described in terms of the medians, means, and standard deviation. Normality was determined using the Kolmogorov–Smirnov test.

To compare the variables of the two groups, the Student's t-test was used for the parametric variable, and the Mann–Whitney U test was used to compare the nonparametric variables. The chi-squared test was used to assess the categorical data.

To understand the role of the independent variables explaining recurrent falls, hierarchically logistic regression was performed, initially introducing the sociodemographic variables (age, race, sex, income, and educational level), followed by some clinical variables (e.g., MMSE, GDS-15, FES-I), the physical performance measurements (TUGT with a cognitive distractor, BBS, and five times sit-to-stand test), and posturographic measures (XSD with EC, VAvg with EC and EO. The significance of the model was set at 0.05%.

## RESULTS

**Figure 1:**
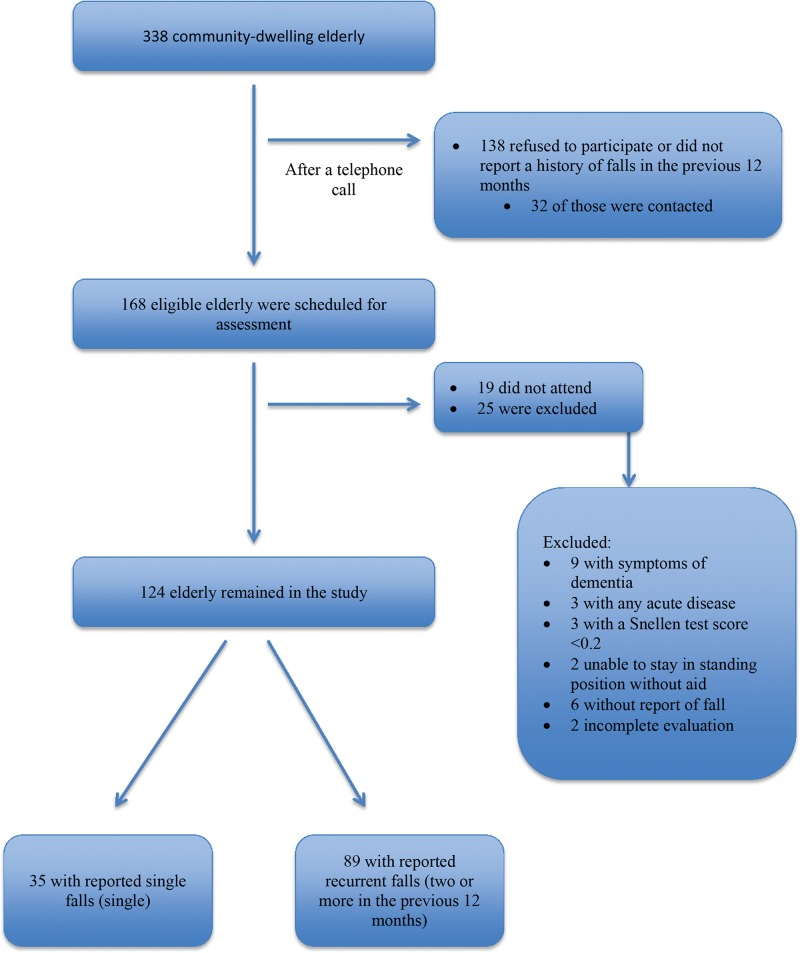
Flow diagram.

The flowchart of the subjects is shown in Figure 1. A total of 338 community-dwelling elderly were screened for participation, and 124 met the inclusion criteria.

Based on the subjects' sociodemographic characteristics, the subjects were predominantly white (67%), female (79%), and married (57%) and had a good cognitive status (MMSE 27) and a total average age of 74 years. According to the subjects' educational level, 40% had less than a primary and 23% had a superior educational level (Table 1). Based on the subjects' income, more than half of the population had an average income, 57% were relatively poor (less than four minimum wages monthly), 39% were considered low and middle class (4–8 minimum wages monthly), and 11% were considered high middle class or rich (>8 minimum wages monthly). There were no statistically significant differences between the single and recurrent fallers groups in terms of their sociodemographic characteristics (Table 1).

Our target population included elderly individuals who experienced falls at least once in the past 12 months prior to the study. Subsequently, the total average number of falls was approximately three times, and among the recurrent fallers, this event was higher, which was four times. Despite the similar clinical conditions between the single and recurrent fallers, the repetitive event among recurrent fallers was significantly associated with higher fear of falling (*p*=0.011) and higher depressive symptoms (*p*=0.007) than the single fallers (Table 1).

A first descriptive analysis of the evaluation tools of the single and recurrent fallers in Table 2 shows that some posturographic measures present better results than the clinical functional tests.

In the top part of Table 2, posturography is measured in terms of the average XSD and the VAvg of the COP (based on three different conditions [EO, EC, and dual tasking]). The most significant result was differentiating recurrent from single fallers in terms of the average XSD EC (*p*=0.011) and the VAvg with EO (*p*=0.014). The dual-tasking condition for both measures was not able to determine the significant differences between the two groups.

In the bottom part of Table 2, the traditional physical performance measurements are not able to differentiate between single and recurrent fallers (TUGT with a cognitive distractor [*p*=0.384, BBS *p*=0.087] and five times sit-to-stand test [*p*=0.548]).

The hierarchical regression was performed in 117 elderly individuals because the seven subjects were unable to perform the five times sit-to-stand test. We used different models controlling the sociodemographic variables. When income and educational level variables were introduced in the model, the results were insignificant to determine the differences between the single and recurrent fallers, considered as an expected finding considering that the Brazilian society has high social inequality. Subsequently, we opted to show here the model controlled by age, sex, and race, which is commonly associated in the literature with higher risk of falls. In this first simple model 1, those variables were also not able to predict the outcome (area under the receiver operating characteristic curve [ROC]=0.63, *p*=0.211) (Table 3).

With the introduction of the clinical variables (medications, psychoactive drugs, FES-I, MMSE, GDS-15) in model 2, we were able to observe significant improvement in the predictability to identify recurrent falls (area under the ROC=0.76, *p*=0.043). This capacity to explain the recurrent falls improved even more once we introduced the measures of the functional tests (TUGT with a cognitive distractor, the BBS, and five time sit-to-stand test) in model 3 (area under the ROC=0.82, *p*=0.031) (Table 3).

Finally, we ran model 4 including all the posturographic variables (XSD EC, Vang EO, Vang EC), significantly improving the explanation regarding recurrent falls. The results were considered unexpected. To confirm this finding, we decided to test each of the posturography variables as alternatives in model 4. By initially controlling XSD EC, a slight gain (1%) in the accuracy of the model was observed, although it was insufficient to explain the difference between the single and recurrent fallers (area under the ROC=0.83, *p*=0.061) (Table 3). When we controlled the last two posturographic variables (VAvg EO and VAvg EC), the results confirmed the general finding; posturography did not improve our understanding regarding the difference between the two groups (Table 3).

After adding all the block variables, Table 3 shows that the high significant independent predictive power in the final adjusted model to explain the difference between the groups can be mostly attributed to the following three variables: depressive symptoms (*p*<0.05), TUGT with a cognitive distractor (*p*<0.05), and the BBS (*p*<0.01).

## DISCUSSION

The main finding of the present study was that posturography did significantly improve the predictive ability to identify recurrent falls. On the contrary, hand functional balance tests (TUGT with a cognitive distractor and the BBS) were considered the recurrent fall predictors in elderly fallers.

We used posturography in our study considering that the etiologies for falls are multifactorial 35. Thus, our goal to compare the quantitative measures of posturography with the most commonly used clinical evaluation test was to establish a new tool that will improve the predictability of falls. The possibility that posturography was more effective in determining recurrent falls compared to the traditional physical performance measurements in our study sample was not confirmed based on the results of the hierarchical regression analysis. Therefore, posturographic measures did not improve the predictive power to identify recurrent fallers, but depressive symptoms and the functional balance tests, such as TUGT with a cognitive distractor and the BBS, were considered the recurrent fall predictors in elderly fallers.

Unfortunately, we do not have any Brazilian study to compare our findings, and subsequently, these findings are discussed in the context of the international literature. The posturographic measures, an increase in the XSD and the VAvg of the COP, are associated with increased fall risk among elderly individuals 13,17,18. In the sample of the Brazilian population, we found that the average XSD EC was associated with the best model for differentiating recurrent from single fallers. This result was consistent with the results of the previous studies, which demonstrated that subjects with a history of falls had increased average lateral displacement with EO and EC 36,37. Our findings also reinforce the idea that visual deprivation during the analysis of the average lateral displacement seems to amplify the difference between the two groups since individuals with postural instability are more dependent on vision than individuals without postural instability 38.

The VAvg of the COP accurately measures postural balance. Our results were performed with EO; hence, differentiating recurrent and non-recurrent fallers was noted, as previously observed by Baloh et al. 39. However, under the logistic regression, this variable did not present an independent predictive power.

The dual-tasking condition predicting the increased risk of falls among elderly individuals was not effective in our study as expected, a result consistent with that of the other studies 30,40, considering that this study had a small sample size for subjects with good cognition and functional capacity. In other words, a second task would reveal impaired balance due to the deterioration of the central capacity or to the prioritization between the two tasks, which were not observed in our study sample.

Although several instruments for assessing mobility and body balance have been developed to predict the risk of falls 24, our results based on the hierarchical logistic regression suggest that there is no single test capable of adequately predicting the risk of falls. In fact, these results were expected considering that the etiologies for falls are multifactorial 35. Even with the addition of posturographic measures, model 4 showed only a marginal improvement (approximately 1%) in predicting single and recurrent fallers. In some manner, it was an expected result considering that falls usually occur under dynamic conditions, specifically in ADLs. To maintain semi-static balance, the sensory information must monitor the body position and adjust the level of joint stiffness to ensure stability, with no significant demands that could lead to falls 25. Moreover, Brazilian studies assessing recurrent falls are insufficient; hence, it is difficult to compare the results of our studies to that of the other studies considering that our study is generally based on study design and protocols, divergences in balance evaluations, and measurement methods.

This study has the following limitations: (1) this study was based on retrospective self-report data regarding fall incidence; (2) subjects voluntarily participated in the study, which implied the recruitment of elderly individuals with good functional capacity from the community-dwelling elderly individuals; (3) non-faller individuals were excluded in the study; and (4) sample size analysis related to the purpose of this study was not performed.

Finally, the clinical implication of this study indicated that the traditional clinical tests, which are simple and economical, were able to predict 82% of a possible faller. When this finding is compared with the 1% posturography contribution to explain the same risks, a question regarding the number of studies required to assess the cost-effectiveness of posturography in assessing recurrent falls has been raised.

In conclusion, our study suggests that additional studies assessing the risk of falls with the use of only one tool are required. Particularly, following up elderly individuals (longitudinal study), including non-fallers, and using new techniques, for example, dynamic posturography, are significantly required in future studies.

Static posturography do not improve the prediction of recurrent and single fallers against other functional or behavioral measurements. On the contrary, TUGT with a cognitive distractor and the BBS presented significant independent predictive power. Thus, we hypothesize that posturography was not considered a recurrent fall predictor and that clinical assessment can be more feasible (low cost and easy to perform) with equal or even better power to predict the incidence of falls in daily clinical practice.

## AUTHOR CONTRIBUTIONS

Cabral KN was responsible for the investigation and manuscript original drafting. Brech GC was responsible for supporting investigation and manuscript writing (review and editing). Alonso AC was responsible for formal analysis and manuscript writing (review and editing). Soares AT was responsible for the conceptualization and funding acquisition. Opaleye DC was responsible for the manuscript original drafting. Greve JMA and Jacob- Filho W were responsible for supervision and manuscript writing.

## Figures and Tables

**Table 1:** Comparison of the subjects' characteristics according to their history of falls.

	Total (N=124)	Single faller (N=35)	Recurrent faller (N=89)	“*p*” value
AGE (YEARS)				
Mean± SD	72.9 (±7.6)	74.4 (±7.4)	72.3 (±7.7)	0.173
60–69	46 (37.1%)	11 (31.4%)	35 (39.3%)	
70–79	47 (37.9%)	13 (37.2%)	34 (38.2%)	
≥80	31 (25.0%)	11 (31.4%)	20 (22.4%)	
GENDER				
Female	98 (79%)	25 (71.4%)	73 (82.0%)	0.289
Male	26 (21%)	10 (28.6%)	16 (18.0%)	
MARITAL STATUS				
Married	71 (57.3%)	20 (57.1%)	51 (57.3%)	0.987
Not married	53 (42.7%)	15 (42.9%)	38 (42.7%)	
RACE				
Caucasian	83 (66.9%)	22 (62.9 %)	61 (68.5 %)	0.545
Not Caucasian	41 (33.1%)	13 (37.1%)	28 (31.5%)	
EDUCATIONAL LEVEL (YEARS)				
0	3 (2.4%)	2 (5.7%)	1 (1.1%)	
1–4	47 (37.9%)	8 (22.9%)	39 (43.8%)	
5–8	29 (23.4%)	11 (31.4%)	18 (20.2%)	0.129
9–11	16 (12.9%)	6 (17.1%)	10 (11.2%)	
≥12	29 (23.4%)	8 (22.9%)	21 (23.6%)	
FAMILY INCOME (minimum wage)				
0 a 2	33 (26.6%)	9 (25.7%)	24 ( 27.0%)	
2.1–4.0	37 (29.8%)	14 (40.0%)	23 (25.8%)	0.202
4.1–6.0	18 (14.5%)	1 (2.9%)	17 (11.1%)	
6.1–8.0	5 (24.0%)	0 (0.0%)	5 (5.6%)	
8.1–9.9	3 (2.4%)	1 (2.9%)	2 (2.2%)	
>10	11 (8.9%)	4 (11.4%)	7 (7.9%)	
FALLS IN THE PREVIOUS YEAR, MEAN ± SD	3.18 (±2.8)	1.0	4.03 (±2.89)	--
FEAR OF FALLING				
Yes	105 (84.7%)	25 (71.4%)	80 (89.9%)	0.010*
No	19 (15.3%)	10 (28.6%)	9 (10.1%)*	
NUMBER OF CHRONIC DISEASES, MEAN ± SD	4.4 (±1.99)	4.2 (±1.86)	4.4 (±2.05)	0.590
MEDICATION USE, MEAN± SD	5.1 (±3.1)	4.8 (±2.8)	5.2 (±3.2)	0.510
USE OF PSHYCOATIVE DRUGS				
Yes	47 (37.9%)	13 (37.1%)	34 (38.2%)	0.913
No	77 (62.1%)	22 (62.9%)	55 (61.8%)	
MMSE^1^, MEAN± SD	27. 1 (±2.25)	27.0 (±2.5)	27.2 (±2.2)	0.728
GDS-15^2^				
Mean ± SD	4.5 (±3.2)	3.3 (±2.0)	5.0 (±3.4)	0.007*
<5 points	73 (58.9%)	26 (74.3%)	47 (52.8%)*	0.029*
≥5 points	51 (41.1%)	9 (25.7%)	42 (47.2%)	
FES- I^3^, MEAN ± SD	30.2 (±7.84)	27.4 (±9.3)	31.3 (±6.9)	0.011*
Snellen <0.5				
Yes	28 (23.1%)	8 (22.9%)	20 (22.7%)	0.860
No	93 (76.9%)	25 (77.1%)	68 (77.3%)	

1 MMSE: Mini-Mental State Examination.

2 GDS-15: 15-item Geriatric Depression Scale.

3 FES- I: Falls Efficacy Scale-International.

N: number of subjects; SD: standard deviation.

* *p*<0.05.

** *p*<0.01.

The data represent the p-values from the Student's t-test, Mann–Whitney U test, and chi-squared test.

**Table 2:** Descriptive statistics for the posturographic measures and physical performance tests.

POSTUROGRAPHIC VARIABLE	TOTAL	SINGLE FALLER	RECURRENT FALLER	“*p*” VALUE
	mean ± SD (N=124)	mean ± SD (N=35)	mean ± SD (N=89)	
XSD^1^ EO	0.149 (±0.324)	0.081 (±0.243)	0.176 (±0.349)	0.153
VAvg^2^ EO	1.238 (±0.676)	1.069 (±0.379)	1.303 (±0.753)	0.014*
XSD^1^ dual tasking	0.415 (±0.199)	0.398 (±0.237)	0.421 ( ±0.184)	0.088
VAvg^2^ dual tasking	1.493 (±0.387)	1.510 (±0.633)	1.470 (±0.575)	0.561
XSD^1^ EC	0.349 (±0.186)	0.289 (±0.115)	0.373 (±0.203)	0.011*
VAvg^2^ EC	1.602 (±1.200)	1.326 (±0.541)	1.71 (±1.365)	0.088
PHYSICAL PERFORMANCE VARIABLE				
TUGT^3^ (seconds)	11.3±4.13	11.1±4.47	11.3±4.02	0.783
TUGT^3^ WITH A COGNITIVE	15.08±6.82	15.94±7.20	14.75±6.69	0.384
DISTRACTOR (seconds)				
BBS^4^	49.02±6.57	50.6±5.12	48.4±6.99	0.087
*FIVE TIMES SIT-TO-STAND TEST*	18.37±6.86	17.67±6.84	18.64±6.89	0.548

Eyes open (EO), eyes closed (EC).

1 Root mean square of the displacement from the center of pressure in the mediolateral axis (XSD).

2 Mean velocity calculated according to the total displacement from the center of pressure in all directions (VAvg).

SD: standard deviation; N: number of subjects.

* *p*<0.05. The data present the p-values from the Mann–Whitney U test.

3 TUGT: timed up and go test.

4 BBS: Berg Balance Scale.

5 IPAQ: International Physical Activity Questionnaire.

N: number of subjects; SD: standard deviation.

The data present the p-values from the Student's t-test and chi-squared test.

**Table 3:** Hierarchical logistic regression model using recurrent falls as the dependent Variable (N=117).

	Variable	Model 1			Model 2			Model 3			Model 4		
		*B*	*SE (B)*	*B*	*B*	*SE (B)*	*B*	*B*	*SE (B)*	*B*	*B*	*SE (B)*	*B*
Age	-0.04	0.03	-0.14	-0.04	0.03	-0.13	-0.07	0.04	-0.21	-0.06	0.04	-0.17
XSD Eyes Closed
	Female	0.80	0.49	0.17	0.91	0.56	0.18	0.88	0.60	0.15	0.97	0.62	0.15
	Caucasian	0.21	0.46	0.05	0.19	0.51	0.04	-0.13	0.57	-0.03	-0.15	0.59	-0.03
	Medication use				0.11	0.09	0.16	0.05	0.10	0.07	0.05	0.11	0.06
	Use of psychoactive drugs				-1.03	0.61	-0.24	-0.82	0.67	-0.16	-0.83	0.69	-0.16
	FES-I^1^				0.05	0.03	0.19	0.05	0.04	0.15	0.05	0.04	0.16
	MMSE^2^				0.07	0.10	0.08	0.13	0.13	0.12	0.13	0.13	0.11
	GDS-15^3^				0.21	0.11	0.33*	0.24	0.12	0.32*	0.26	0.12	0.32*
	TUGT with a cognitive distractor^4^							-0.14	0.07	-0.35*	-0.15	0.07	-0.36*
	BBS^5^							-0.31	0.11	-0.61**	-0.29	0.12	-0.55*
	Five times sit-to-stand test							-0.03	0.04	-0.10	-0.05	0.04	-0.12
	X SD EC^6^										3.70	1.97	-0.27
	Area under the ROC	**0.63**			**0.76**			**0.82**			**0.83**		
	Wald chi-squared test of variance†	Wald chi^2^ (3)=4.40***p*=0.211**			Wald chi^2^ (5)=11.46***p*=0.043**			Wald chi^2^ (3)=8.87***p*=0.031**			Wald chi^2^ (1)=3.51***p*=0.061**		

	Variable	Model 1			Model 2			Model 3			Model 4		
		*B*	*SE (B)*	*B*	*B*	*SE (B)*	*B*	*B*	*SE (B)*	*B*	*B*	*SE (B)*	*B*
Age	-0.04	0.03	-0.14	-0.04	0.03	-0.13	-0.07	0.04	-0.21	-0.05	0.05	-0.14
VAvg Eyes Open
	Female	0.80	0.49	0.17	0.91	0.56	0.18	0.88	0.60	0.15	0.90	0.62	0.14
	Caucasian	0.21	0.46	0.05	0.19	0.51	0.04	-0.13	0.57	-0.03	-0.27	0.59	-0.05
	Medication use				0.11	0.09	0.16	0.05	0.10	0.07	0.01	0.11	0.02
	Use of psychoactive drugs				-1.03	0.61	-0.24	-0.82	0.67	-0.16	-0.62	0.70	-0.12
	FES-I^1^				0.05	0.03	0.19	0.05	0.04	0.15	0.05	0.04	0.16
	MMSE^2^				0.07	0.10	0.08	0.13	0.13	0.12	0.13	0.13	0.11
	GDS-15^3^				0.21	0.11	0.33*	0.24	0.12	0.32*	0.28	0.12	0.35*
	TUGT with a cognitive distractor^4^							-0.14	0.07	-0.35*	-0.16	0.07	-0.39*
	BBS^5^							-0.31	0.11	-0.61**	-0.28	0.11	-0.53*
	Five times sit-to-stand test							-0.03	0.04	-0.10	-0.03	0.04	-0.08
	VAvg EO^7^										1.02	0.68	0.27
	Area under the ROC	**0.63**			**0.76**			**0.82**			**0.82**		
	Wald Chi-squared test of variance†	Wald chi^2^ (3)=4.40***p*=0.211**			Wald chi^2^ (5)=11.46***p*=0.043**			Wald chi^2^ (3)=8.87***p*=0.0311**			Wald chi^2^ (1)=2.73***p*=0.130**		

	Variable	Model 1			Model 2			Model 3			Model 4		
		*B*	*SE (B)*	*B*	*B*	*SE (B)*	*B*	*B*	*SE (B)*	*B*	*B*	*SE (B)*	*B*
Age	-0.04	0.03	-0.14	-0.04	0.03	-0.13	-0.07	0.04	-0.21	-0.05	0.05	-0.14
VAvg Eyes Closed
	Female	0.80	0.49	0.17	0.91	0.56	0.18	0.88	0.60	0..15	0.97	0.61	0.15
	Caucasian	0.21	0.46	0.05	0.19	0.51	0.04	-0.13	0.57	-0.03	-0.30	0.60	-0.05
	Medication use				0.11	0.09	0.16	0.05	0.10	0.07	0.02	0.11	0.02
	Use of psychoactive drugs				-1.03	0.61	-0.24	-0.82	0.67	-0.16	-0.63	0.69	-0.12
	FES-I^1^				0.05	0.03	0.19	0.05	0.04	0.15	0.05	0.04	0.16
	MMSE^2^				0.07	0.10	0.08	0.13	0.13	0.12	0.13	0.13	0.12
	GDS-15^3^				0.21	0.11	0.33*	0.24	0.12	0.32*	0.26	0.12	0.32*
	TUGT with a cognitive distractor^4^							-0.14	0.07	-0.35*	-0.15	0.07	-0.38*
	BBS^5^							-0.31	0.11	-0.61**	-0.29	0.11	-0.56**
	Five times sit-to-stand test							-0.03	0.04	-0.10	-0.03	0.04	-0.09
	VAvg EC^8^										0.54	0.45	0.25
	Area under the ROC	**0.63**			**0.76**			**0.82**			**0.82**		
	Wald chi-squared test of variance†	Wald chi^2^ (3)=4.40***p*=0.211**			Wald chi^2^ (5)=11.46***p*=0.043**			Wald chi^2^ (3)=8.87***p*=0.0311**			Wald chi^2^ (1)=1.42***p*=0.234**		

† Whether the addition of the corresponding block of Variables improves the predictive power of the model, with *p*<0.05 indicating a significant increase.

*B*, beta coefficient; SE (B), standard error of the beta coefficient; β, standardized beta coefficient.

1 FES-I: Falls Efficacy Scale-International.

2 MMSE: Mini-Mental State Examination.

3 GDS-15: 15-item Geriatric Depression Scale.

4 TUGT: Timed up and go test with a cognitive distractor.

5 BBS: Berg Balance Scale.

6 XSD EC: Average amplitude of displacement of the center of pressure in the mediolateral plane in the eyes closed condition.

7 VAvg EO: Average speed calculated by the total COP displacement in all directions, expressed in cm/s in the eyes opened condition.

8 VAvg EC: Average speed calculated by the total COP displacement in all directions, expressed in cm/s in the eyes closed condition.

N: Number of subjects.

* *p*<0.05.

** *p*<0.01.

*** *p*<0.001.

N: number of subjects.

* *p*<0.05.

** *p*<0.01.

*** *p*<0.00.
